# Solid-State Characterization and Compatibility Studies of Penciclovir, Lysine Hydrochloride, and Pharmaceutical Excipients

**DOI:** 10.3390/ma12193154

**Published:** 2019-09-27

**Authors:** Rafaela Z. C. Meira, Isabela F. B. Biscaia, Camila Nogueira, Fabio S. Murakami, Larissa S. Bernardi, Paulo R. Oliveira

**Affiliations:** 1 Post Graduation Program in Pharmaceutical Sciences, Department of Pharmacy, Universidade Estadual do Centro-Oeste/UNICENTRO, Guarapuava, PR 85040-080, Brazil; 2 Department of Pharmacy, Federal University of Paraná, Av. Prefeito Lothário Meissner, 632, Jardim Botânico, Curitiba, PR 80210-170, Brazil

**Keywords:** penciclovir, lysine, compatibility, characterization, excipients, differential scanning calorimetry, thermogravimetry

## Abstract

The physical and chemical characterization of the solid-state properties of drugs and excipients is fundamental for planning new formulations and developing new strategies for the treatment of diseases. Techniques such as differential scanning calorimetry, thermogravimetry, X-ray powder diffraction, Fourier transform infrared spectroscopy, and scanning electron microscopy are among the most commonly used techniques for these purposes. Penciclovir and lysine are individually used to treat the herpes virus. As such, the development of a formulation containing both drugs may have therapeutic potential. Solid-state characterization showed that both penciclovir and lysine were crystalline materials with melting points at 278.27 °C and 260.91 °C, respectively. Compatibility studies of penciclovir and lysine indicated a possible interaction between these substances, as evidenced by a single melting point at 253.10 °C. The compatibility of several excipients, including ethylenediaminetetraacetic acid, cetostearyl alcohol, sodium lauryl sulphate, di-tert-butyl methyl phenol, liquid petrolatum, methylparaben, nonionic wax, paraffin, propylene glycol, and propylparaben, was evaluated in ternary (penciclovir-lysine-excipient) mixtures (1:1:1, *w*/*w*/*w*) to determine the optimal formulation. The developed formulation was stable under accelerated and ambient conditions, which demonstrated that the interaction between penciclovir and lysine was suitable for the development of a formulation containing both drugs.

## 1. Introduction

**Figure 1:**
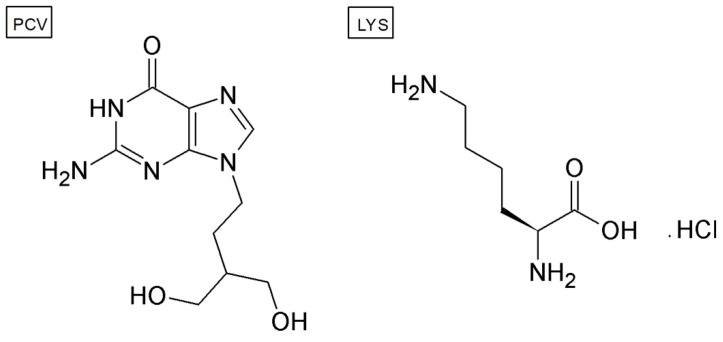
Molecular structure of penciclovir (PCV) and lysine (LYS).

Penciclovir (PCV, 9-[4-hydroxy-3-(hydroxymethyl)butyl]guanine, Figure 1), a synthetic acyclic guanine derivative, is a potent antiviral drug with activity against herpes simplex virus, varicella zoster virus, Epstein–Barr virus, hepatitis virus, and cytomegalovirus [1,2,3]. Penciclovir is converted to penciclovir triphosphate by thymidine kinase and cellular phosphokinase, and its phosphorylated form inhibits virus DNA polymerase and viral replication [3,4].

PCV is widely indicated for the treatment of recurrent cold sores caused by the herpes virus [5]. Compared to acyclovir, PCV is more stable, resulting in greater in vitro antiviral activity and a longer half-life in vivo [6,7]. PCV can be formulated in topical preparations [8,9,10,11] but is absorbed poorly via the oral route. As such, its prodrug (famciclovir) is used for oral administration.

The antiviral potential of an important amino acid, L-lysine (LYS, 2,6-diaminocaproic acid, Figure 1), suggests that new strategies could be developed to treat recurrent herpes [10,12]. Since LYS is extremely unstable under normal conditions, it is formulated as lysine hydrochloride [13]. LYS reduces viral replication by antagonising the effects of arginine, which is essential for viral protein synthesis [14,15].

The development of new formulations must account for variability in the properties of the materials used. Specifically, variability between manufacturers must be considered. Thus, a solid-state characterization of active pharmaceutical ingredients and an evaluation of compatibility between the drug and excipients is essential [16,17]. Characterization and compatibility studies use a variety of techniques to evaluate the physical characteristics of formulations. The most common techniques used are differential scanning calorimetry (DSC), thermogravimetry (TG), X-ray powder diffraction (XRPD), infrared Fourier transform spectroscopy (FTIR), and scanning electron microscopy (SEM) [18,19].

Compatibility studies are important for prediction of the stability, chemical properties, efficacy, and safety of formulations. Compatibility is typically evaluated during preformulation studies, using binary and ternary systems of drugs and excipients [20,21,22,23]. Compatibility studies may identify important changes in a drug upon formulation, such as changes in melting point or glass transition. The effects of these changes can be characterized using DSC, TG, XPRD, FTIR and SEM [24]. Following the development of the proposed finished product, stability studies are required to ensure the quality of the product and to determine the shelf life and storage conditions [25].

The most used excipients in topical formulations containing nucleoside analogues available in the Brazilian market are ethylenediamine tetra-acetic acid (chelating agents), methylparaben and propylparaben (preservatives), di-tert-butyl methyl phenol (antioxidants), sodium lauryl sulphate (surfactants), liquid petrolatum and paraffin (viscosity donors), cetostearyl alcohol and nonionic wax (viscosity donors and surfactants), and propylene glycol (solvent, preservative and humectant) [26].

The objective of this study was to perform a physical–chemical characterization of PCV and LYS, and to evaluate the chemical compatibility of these molecules in ternary mixtures with excipients for the future development of a new topical pharmaceutical formulation.

## 2. Materials and Methods 

### 2.1. Materials

Drugs and excipients included Penciclovir (kindly donated by EMS Pharma Ltd., Hortolândia, SP, Brazil), lysine hydrochloride (Infinity Pharma Ltd., Campinas, SP, Brazil), ethylenediamine tetra-acetic acid (EDTA) (Anidrol, Diadema, SP, Brazil), cetostearyl alcohol (CET ALC) (Êxodo Científica, Sumaré, SP, Brazil), sodium lauryl sulphate (LAURYL) (Anidrol, Diadema, SP, Brazil), di-tert-butyl methyl phenol (BHT) (Synth, Diadema, SP, Brazil), liquid petrolatum (LIQ PET) (Êxodo Científica, Sumaré, SP, Brazil), methylparaben (METHYL) (Êxodo Científica, Sumaré, SP, Brazil), nonionic wax (POLAWAX) (Êxodo Científica, Sumaré, SP, Brazil), paraffin (PARAFF) (Vetec, Rio de Janeiro, Brazil), propylene glycol (PROP GLY) (Synth Ltd., Diadema, SP, Brazil), and propylparaben (PROPYL) (Êxodo Científica, Sumaré, SP, Brazil). For the chromatographic analysis: ultrapure water (Milli-Q^®^, Millipore, Burlington, MA, USA), methanol (Tedia, Fairfield, OH, USA), derivatization reagent composed of orthophthaldehyde (Sigma-Aldrich Ltd., St. Louis, MO, USA), 2-mercaptoethanol (Sigma-Aldrich Ltd., St. Louis, MO, USA), and boric acid (Êxodo Científica, Sumaré, SP, Brazil).

### 2.2. Physical–Chemical Characterization 

#### 2.2.1. Differential Scanning Calorimetry (DSC)/Thermogravimetry (TG)

Pure PCV, LYS, and excipients used in the development of the formulation were analyzed individually, and in binary (PCV:LYS 1:1, *w*/*w*) and ternary mixtures (PCV:LYS:Excipient, 1:1:1, *w*/*w*/*w*). Samples were analyzed using simultaneous DSC/TG equipment (SDT-Q600^®^ TA Instruments, Tokyo, Japan). The samples were placed in aluminium crucibles (about 4 mg of sample). The temperature range used was 20–400 °C, with a heating rate of 10 °C min^−1^, under a nitrogen atmosphere (100 mL min^−1^).

#### 2.2.2. X-ray Powder Diffraction (XRPD)

The X-ray powder diffraction patterns were obtained using a D2 Phaser^®^ Bruker diffractometer (Billerica, MA, USA). Samples were analysed across the range of 6–40° (2θ), with a step time of 1 s and increment of 0.05° using CuKa tube. Samples analysed included pure drugs (PCV and LYS), and 1:1 (*w*/*w*) mixtures of PCV and LYS. To avoid preferential orientation, the samples were carefully placed over the sample holder and maintained at 5 rpm during the analyses.

#### 2.2.3. Diffuse Reflectance Fourier Transform Infrared Spectroscopy (FTIR)

The FTIR spectra (Frontier^®^ Perkin Elmer, Waltham, USA) acquired for the pure drugs (PCV and LYS) and for the binary mixtures were obtained across a scan range of 4000–600 cm^−1^, averaged across more than 32 scans, with a spectral resolution of 4 cm^−1^. Spectral information was obtained using diffuse reflection, based on the incidence and reflection of light powder, defined as a sufficient quantity to cover the sample support disk.

#### 2.2.4. Scanning Electron Microscopy (SEM)

Photomicrographs of the pure drugs (PCV and LYS) and the mixture 1:1 (*w*/*w*) were obtained using a microscope (VEGA 3 SB^®^ TESCAN, Brno, Czech Republic) with a secondary electron detector (SE) (Everhart–Thornley type) for high vacuum, with a positive potential front grille and a 30 kV voltage filament. The samples were mounted on an aluminium support and fixed on double-sided carbon adhesive tape.

### 2.3. Compatibility Studies

For the compatibility analysis of PCV, LYS, and excipients, ternary systems at a ratio of 1:1:1 (*w*/*w*/*w*) were evaluated. The compatibility of the ternary mixtures was determined using DSC and TG as previously described in Section 2.2.1. Mixtures were prepared by weighing 50 mg of each substance into a small plastic vial (Eppendorf^®^, Hamburg, Germany). The components were vortex mixed (Quimis^®^, Diadema, SP, Brazil) for 1 min at 2500 rpm. This mixing procedure promoted homogeneity [27].

### 2.4. Selection of Excipients and Development of Formulation

We evaluated excipients used in the commercial cream formulations that contain penciclovir and acyclovir. The cream was chosen based on previous studies [28,29]. The components in the aqueous phase and the oil phase were preheated, then removed from the heat source once they reached the desired temperature. The mixtures were then allowed to cool at room temperature with constant stirring. The formulation cannot be further described, as it is currently in the initial phases of the patent process.

### 2.5. Stability Study

Accelerated stability was evaluated at 40 °C ± 2 °C with 75% ± 5% relative humidity for 6 months. In addition, stability was evaluated at ambient laboratory conditions (25 °C ± 2 °C with 75% ± 5% relative humidity) for the same period, in accordance with International Conference on Harmonisation (ICH) Guidance [30]. A stability-indicating HPLC method was developed and validated to evaluate stability. A Shimadzu LC-20A Prominence chromatographic system (Shimadzu, Kyoto, Japan) was used with the following chromatographic conditions: Phenomenex (Torrance, CA, USA) Luna C_18_ column (150 mm × 4.60 mm, 5 μm) and PDA detection. The mobile phase elution (1 mL min^−1^) was performed in isocratic mode for PCV, with a ratio of 20:80 (methanol:water, v/v), and linear gradient for LYS, starting at a ratio of 50:50 (methanol:water, v/v) and ending at 80:20 (v/v). For the determination of LYS, the samples needed to be derivatized using orthophthaldehyde and 2-mercaptoethanol. PCV and LYS were evaluated, respectively, in the following wavelengths: 254 nm and 227 nm.

## 3. Results and Discussion

### 3.1. Characterization of Penciclovir and Lysine and the Binary Mixture Compatibility Studies

**Figure 2:**
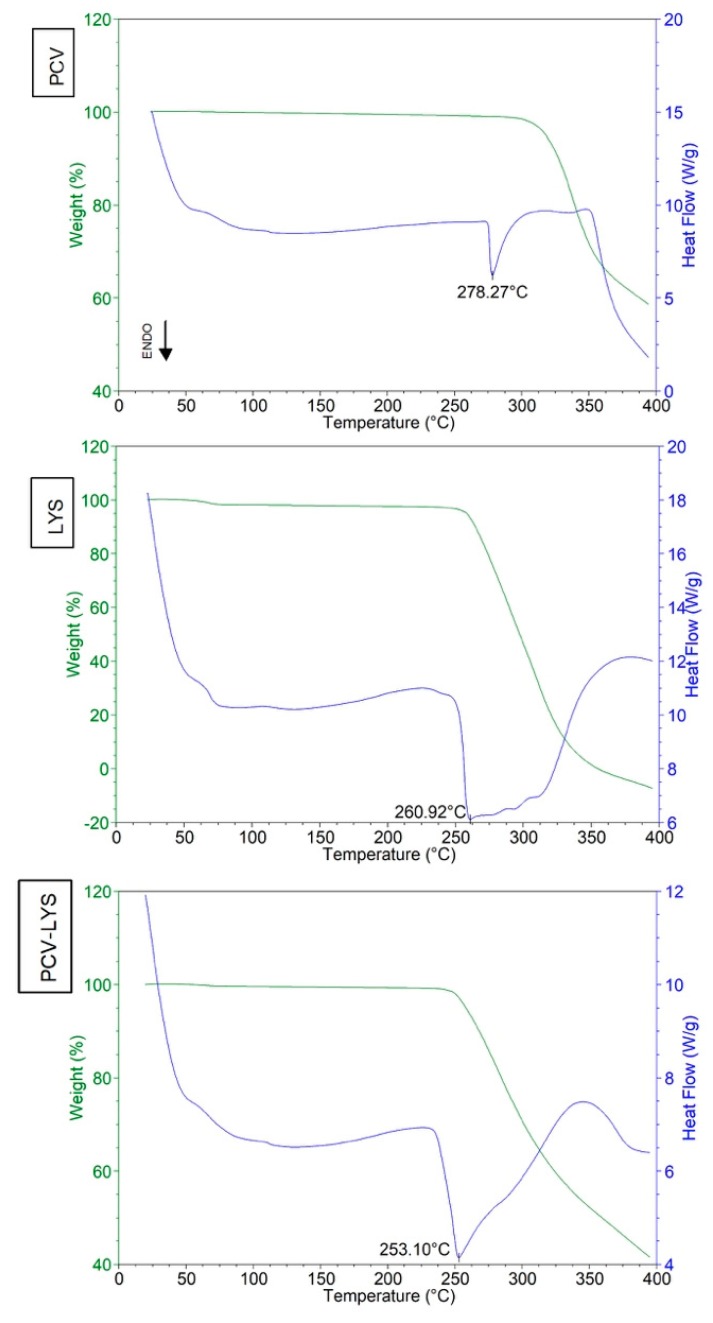
Differential scanning calorimetry (DSC) curves of PCV, LYS, and PCV-LYS mixture.

The DSC curve of PCV (Figure 2) showed an endothermic peak at 278 °C (ΔH_fusion_ 175.2 J g^−1^), which indicated its melting temperature [31]. At 300 °C, the TG curve showed the beginning of the degradation of the PCV. The DSC curve of LYS (Figure 2) showed an endothermic peak at 261 °C, which corresponded to its melting temperature and degradation (as observed in the TG curve), which agreed with a previous study [32]. A mass loss of 1.66% at about 70 °C was observed due to the loss of water.

The differential scanning calorimetry analysis of the binary physical mixture containing PCV and LYS (Figure 2) showed an endothermic event at 253 °C. This was the result of a reduction in the melting point of the LYS when combined with the PCV, which agreed with a previous study [33]. The disappearance of the PCV melting event suggested that the PCV may have interacted strongly with the LYS. This melting point information was not indicative of incompatibility, as the manufacturing process does not reach 253 °C. The interaction between PCV and LYS was further evaluated using complementary methods.

**Figure 3:**
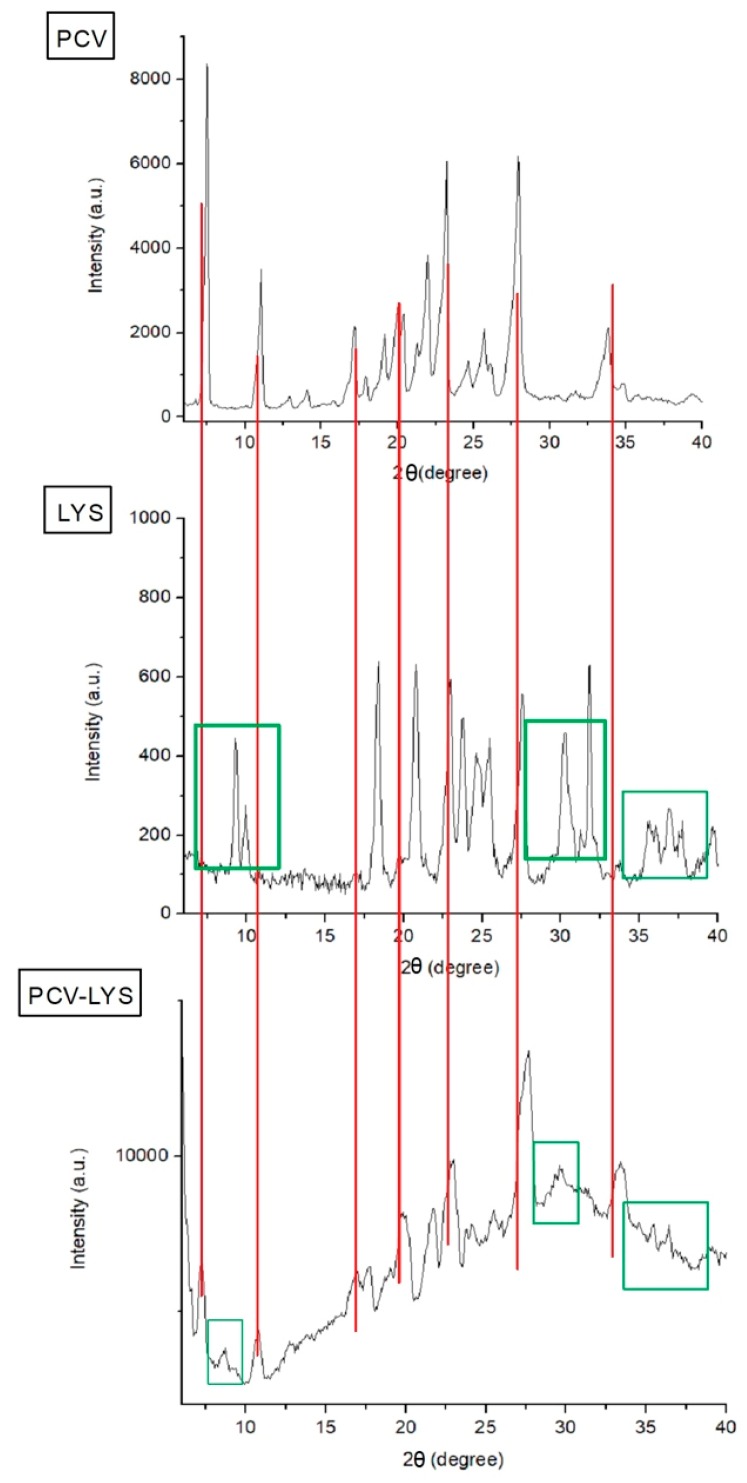
X-ray powder diffraction (XRPD) analysis of PCV, LYS and PCV-LYS.

Analysis using XRPD showed that PCV had diffraction peaks at angles of 8°, 11°, 17°, 24°, 28°, and 34°, which agree with the values for PCV as a crystalline orthorhombic system [31,34]. The LYS diffractogram, also presented in Figure 3, showed peaks at 10°, 17°, 21°, 25°, 31°, and 39°. The LYS peak intensities were lower than those of PCV, but at angles similar to those described by Batista and Kasten et al., Lysine exists in a monoclinic crystallographic system, with four molecules per unit cell [33,35]. Since LYS is hygroscopic, some differences may be observed between analyses [36].

The XRPD patterns obtained for each drug and for the physical mixture (Figure 3) showed that the main crystallographic peaks for PCV (lines in red) and LYS (green squares, as well as peaks overlain by reason of low intensity) were maintained, demonstrating that each compound was intact [37].

**Figure 4:**
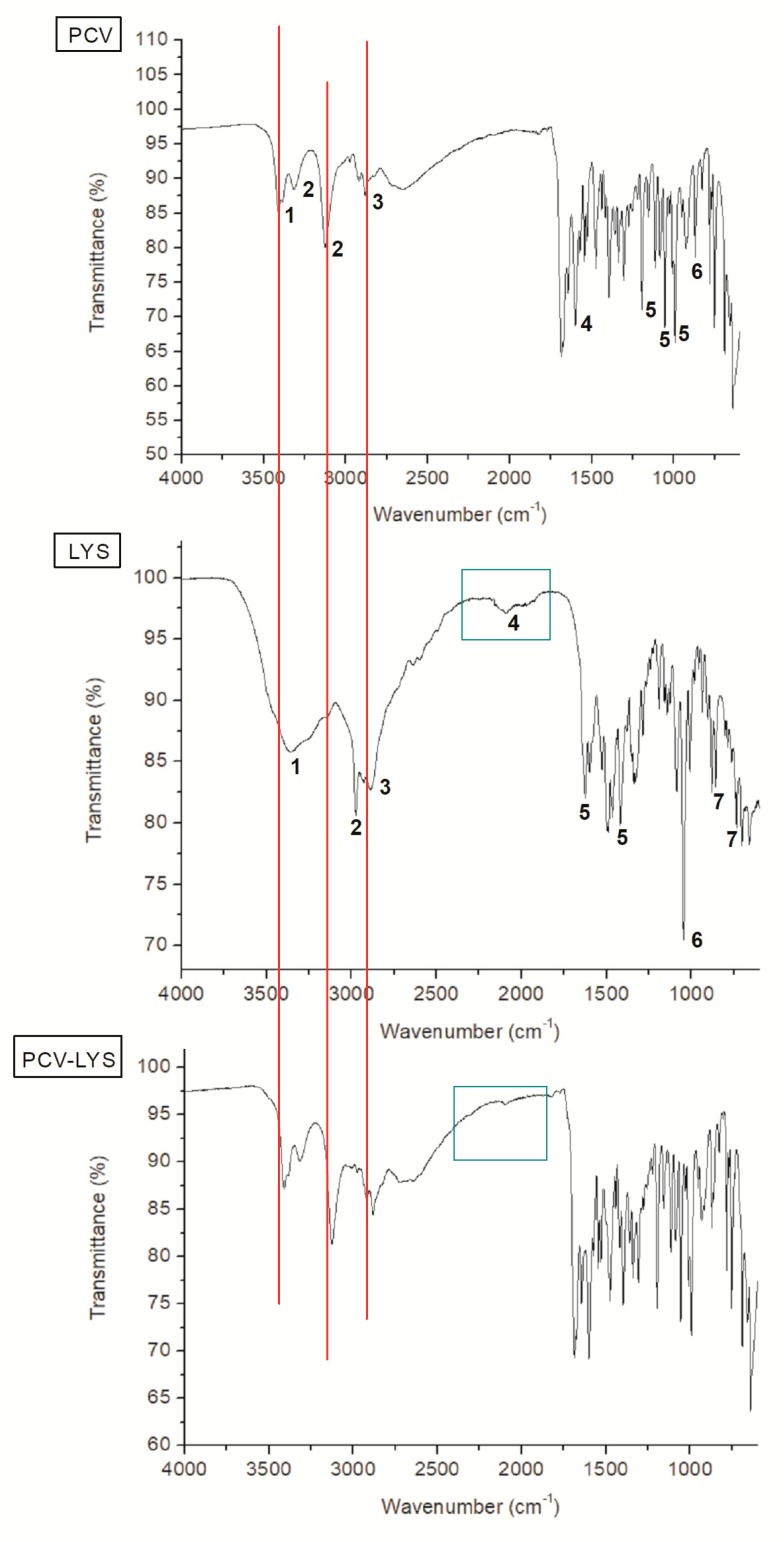
Infrared Fourier transform spectroscopy (FTIR) spectra of PCV, LYS and PCV-LYS.

Penciclovir contains several functional groups that were detected using FTIR (Figure 4). Peaks in the absorption region of 3400 cm^−1^ corresponded to the aliphatic amine of PCV. Peaks at 3313 cm^−1^ and 3126 cm^−1^ corresponded to stretching vibrations of the N–H bonds. The peak at 2885 cm^−1^ corresponded to symmetrical vibrations of the CH_2_ group. The absorption band at 1683 cm^−1^ corresponded to the C=O of the amide moiety. Stretch vibrations were observed at 1381 cm^−1^, 1310 cm^−1^, and 1176 cm^−1^, which corresponded to the overlap of the C–O and C–N bonds, which were related to amides and amines. In the 848 cm^−1^ absorption region, off-plane folds corresponded to C–H bonds [38]. The spectrum agreed with previously reported spectra [39]. Nucleoside analogues have common vibration regions, as demonstrated by the acyclovir FTIR spectra reported by Akimsheva et al. (2019), including amines and cyclic amides [40]. Table 1 assists in understanding the analysis performed.

The spectrum of LYS (Figure 4, Table 1) contained a peak at 3366 cm^−1^, which corresponded to the stretching vibration (H–O–H) of the water present associated with the molecule. At 3087 cm^−1^, a shoulder corresponding to the stretching vibrations of the C–H bonds was observed in proximity to a peak from 3000 cm^−1^ to 2800 cm^−1^ which corresponded to the N–H bonds of the NH_2_ moiety. A possible angular deformation was observed for the NH_2_ moiety between 2200 cm^−1^ and 1900 cm^−1^. Absorption at 1624 cm^−1^ corresponded to the stretching vibrations of the C=O bond. The band at 1425 cm^–1^ may correspond to symmetrical vibrations of the carboxylate group. The band at 1045 cm^−1^ corresponded to vibrations of the C–N bond. Deformations of the vibrations of the carboxylate group corresponded to absorption bands at 735 cm^−1^, 705 cm^−1^, and 663 cm^−1^, overlapping the absorption records of H–Cl [38]. These results agree with previous reports [32,41].

The FTIR of the physical mixture of PCV and LYS (Figure 4) showed a superposition of the spectra of each individual component, which demonstrated that there was no interaction between the drugs. Although some peaks overlapped due to similar chemical moieties, the absorption bands that corresponded to specific moieties were unchanged (Figure 4, PCV—red lines; LYS—green squares).

**Figure 5:**
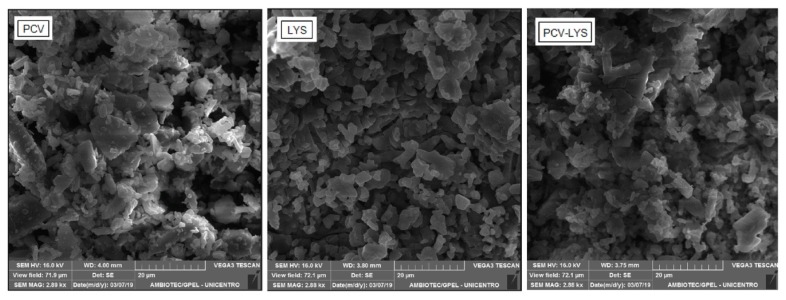
Photomicrographs of PCV, LYS, and PCV-LYS using scanning electron microscopy (SEM) (2880×).

In Figure 5, the morphologies are shown. The binary mixture (PCV-LYS) was not different morphologically from each individual component. This result further supported that there was no interaction between the two drugs.

### 3.2. Compatibility Study in Ternary Mixtures

For the compatibility study, the excipients present in most commercial formulations that contain acyclovir and penciclovir were evaluated. As described by Bruni et al. (2010), analysis of binary and ternary mixtures resulted in more reliable results using techniques such as DSC [42]. Ternary mixtures were used because the binary mixture had a different melting point than the individual components.

The T_peak_ temperatures of the main event, which corresponded to the fusion temperature of PCV and LYS, and the binary and ternary mixtures are summarized in Table 2. Typically, the ΔH_fusion_ is also evaluated. However, all events were very broad, resulting in values that were not comparable between mixtures. The DSC curve of each individual excipient is shown in the supplementary materials Section.

**Figure 6:**
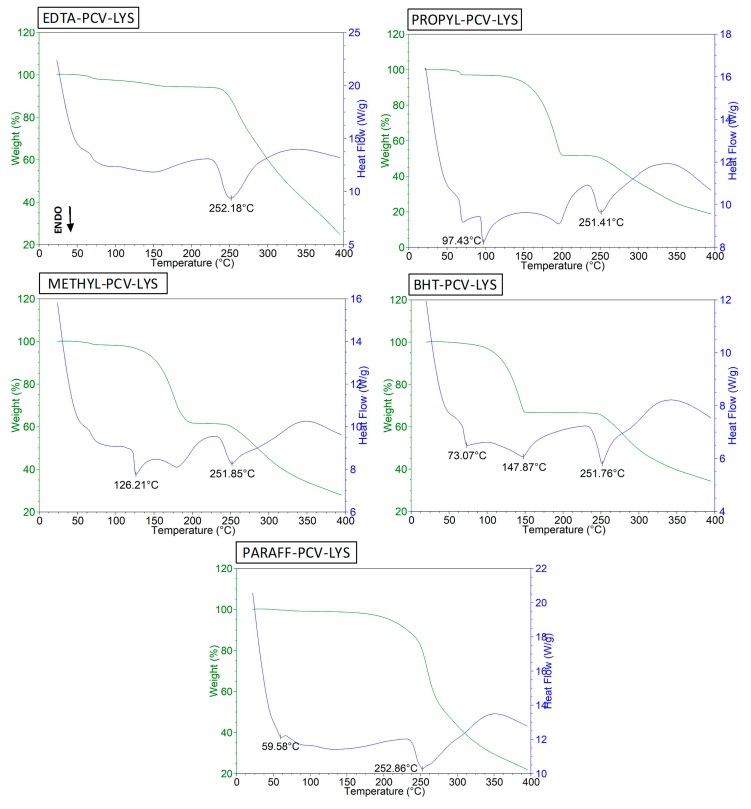
DSC curves of EDTA-PCV-LYS, PROPYL-PCV-LYS, METHYL-PCV-LYS, BHT-PCV-LYS and PARAFF-PCV-LYS.

The DSC curve of the EDTA (supplementary materials, Figure S1) revealed its melting point to be 253 °C, which agreed with previous reports [26]. In the ternary mixture (Figure 6, EDTA-PCV-LYS), the endothermic events overlapped, and extensive degradation was observed, which was similar to the effects observed in the individual DSC profiles. These results suggest that there was no interaction between the EDTA and the drugs.

The DSC curve of the PROPYL had a melting event at 97 °C [43] and degradation occurred at 215 °C (Figure S1). For the METHYL, the melting point was 126 °C (Figure S1), which agrees with previous reports [26,44], and degradation occurred at approximately 196 °C. The ternary system (Figure 6, PROPYL-PCV-LYS and METHIL-PCV-LYS) of these compounds was a superposition of each individual preservative curve and the PCV-LYS curve, with all events separate and well defined, which indicated that there was no interaction between the compounds.

BHT melted at 71 °C in our study (Figure S1), which agreed with previous reports [26,45]. The melting point for the BHT in the ternary mixture (Figure 6, BHT-PCV-LYS) did not change, which suggested that there was no interaction between these compounds.

Paraffin has a melting point between 50 and 61 °C, as evidenced by an endothermic peak in the DSC/TG analysis of its pure form (Figure S1) and degrades at about 268 °C [26]. In the mixture (PARAFF-PCV-LYS), we showed (Figure 6) that the endothermic peak for PCV-LYS was maintained, and the endothermic peak for paraffin was also unchanged. All components degraded near 253 °C, which suggests that there was no interaction between the drugs and the excipient.

**Figure 7:**
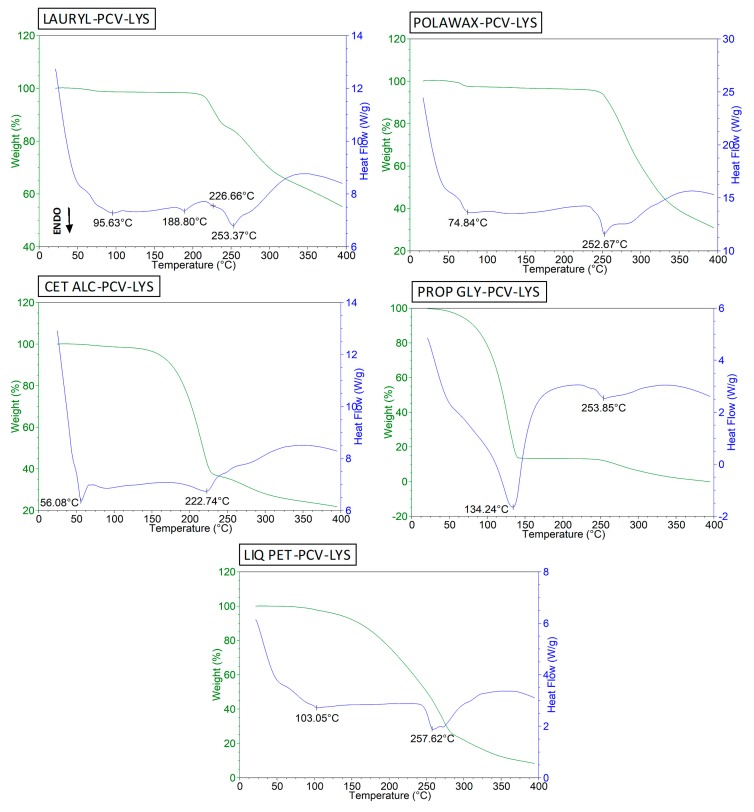
DSC curves for LAURYL-PCV-LYS, POLAWAX-PCV-LYS, CET ALC-PCV-LYS, PROP GLY-PCV-LYS and LIQ PET-PCV-LYS.

The thermal behaviour of LAURYL is shown in Figure S2 in the supplementary materials. We observed peaks near 200 °C and 260 °C, which agreed with the results of previous studies [46,47]. The event at 95 °C may have been due to the presence of water. Analysis of the ternary mixture (Figure 7, LAURYL-PCV-LYS) resulted in distinct peaks that corresponded to the endothermic events of the LAURYL and PCV-LYS, which indicated that there was no interaction between these compounds.

The melting point of the POLAWAX (Figure S2) was about 55 °C, which agreed with previous results [26]. The ternary mixture with PCV and LYS exhibited the same thermal behaviours of the individual components (Figure 7).

The melting point of the CET ALC was observed at 56 °C (Figure S2) [26]. At about 222 °C, degradation was observed. In the ternary CET ALC-PCV-LYS mixture (Figure 7), the drug melting peak at 253 °C disappeared, which indicated complete solubilization of the PVC and LYS in the CET ALC. This result agreed with previous reports that showed strong interactions between CET ALC and prednicarbate [43], lapachol [48], and ibuprofen [49]. However, since CET ALC is a surfactant, solubilisation of the drugs in melted CET ALC does not necessarily represent incompatibility [43,48,49].

The melting point of the PROP GLY was 144 °C (Figure S2), which is in accordance with the literature [50]. The ternary mixture with PCV-LYS (Figure 7, PROP GLY-PCV-LYS) resulted in a 10 °C decrease in the melting point, but the event at 253 °C did not change. A decrease in the intensity of the PCV-LYS event was observed, which may have been due to partial solubilisation of the drugs in the PROP GLY. This result agreed with a result from a previous study [50].

The thermal profile of liquid paraffin (LIQ PET) is shown in Figure S2, which contained a large degradation event, similar to that observed in the literature [43]. In the LIQ PET-PCV-LYS curve (Figure 7), the PCV-LYS event shifted from 253 to 257 °C. This small change caused by the LIQ PET was also observed in a binary mixture with prednicarbate [43].

The possible interaction of the drugs with some of the excipients, such as PROPYL, METHYL, and POLAWAX, may be related to the presence of hydroxyl and amine groups, which is more exposed in both drug and excipient molecules. This probable interaction was also reported by Silva and Cavalheiro (2015) in a thermal analysis performed with alginate and monoethanolamine [51].

The selection of the excipients is a fundamental step in the development of pharmaceutical products. This process requires compatibility studies to justify the choice of each component [52]. Based on this study and the functions they perform, we included paraffin, lauryl sodium sulfate, cetostearyl alcohol, propylene glycol, and liquid petrolatum in our formulation.

The compatibility analysis between drugs and excipients performed by a ternary system proved to be efficient in the evaluation of formulations consisting of two active pharmaceutical ingredients, optimizing the number of tests and providing results closer to the real formulation components.

### 3.3. Stability Studies

After the selection of excipients, the stability of the developed O/W emulsion was evaluated under accelerated and ambient conditions. Both penciclovir and lysine remained stable, as demonstrated in Table 3. Furthermore, neither the colour nor the odour of the formulation changed over time.

Analysis of the binary PCV-LYS mixture using DSC suggested an interaction between these drugs. No interaction was observed using FTIR, XRPD, or SEM. Our stability study further confirmed that the interaction between the PCV and LYS was not an incompatibility. This information supports the stability, efficacy, and safety of the developed formulation.

## 4. Conclusions

The physicochemical characterization and compatibility studies of the drugs and excipients present in a formulation are an important step in the development of pharmaceutical formulations. Penciclovir and lysine were characterized by DSC, TG, XRPD, FTIR, and SEM. Thermal analyses were used to identify possible incompatibilities, which aided in choosing formulation components. The interaction observed between the PCV and LYS using DSC/TG was not apparent using any other techniques. Thus, this interaction was not considered an incompatibility, and the final product was considered safe and stable.

## 5. Patents

Patent required, number BR102019015154-4.

## Figures and Tables

**Table 1:** Position and assignment of PCV and LYS FTIR bands.

**PCV**		
**Peak**	**Bonding**	**Absorption Region (cm^−1^)**
1	N–H _(aliphatic)_	3400
2	N–H _(cyclic)_	3313/3126
3	CH_2 (symmetrical vibration)_	2885
4	C=O	1683
5	C–O and C–N	1381/1310/1176
6	C–H _(off-plane folds)_	848
**LYS**		
**Peak**	**Bonding**	**Absorption Region (cm^−1^)**
1	H–O–H	3366
2	C–H	3087
3	N–H	3000-2800
4	NH_2_	2200/1900
5	C=O	1624/1425
6	C–N	1045
7	H–Cl and C=O _(deformations)_	735/705/663

**Table 2:** T_peak_ temperatures of the main event in the DSC curves of PCV, LYS, and binary and ternary mixtures. Ethylenediamine tetra-acetic acid (EDTA), cetostearyl alcohol (CET ALC), sodium lauryl sulphate (LAURYL), di-tert-butyl methyl phenol (BHT), liquid petrolatum (LIQ PET), methylparaben (METHYL), nonionic wax (POLAWAX), paraffin (PARAFF), propylene glycol (PROP GLY), propylparaben (PROPYL).

Sample	Ratio (Drug:Drug or Drug:Drug: Excipient)	T_peak_/°C
PCV	-	278.27
LYS	-	260.92
PCV-LYS	1:1	253.10
EDTA-PCV-LYS	1:1:1	252.18
PROPYL-PCV-LYS	1:1:1	251.41
METHYL-PCV-LYS	1:1:1	251.85
BHT-PCV-LYS	1:1:1	251.76
PARAFF-PCV-LYS	1:1:1	252.86
LAURYL-PCV-LYS	1:1:1	253.37
POLAWAX-PCV-LYS	1:1:1	252.67
CET ALC-PCV-LYS	1:1:1	222.74
PROP GLY-PCV-LYS	1:1:1	253.85
LIQ PET-PCV-LYS	1:1:1	257.62

**Table 3:** Analysis of PVC-LYS content in formulations subjected to accelerated stability conditions.

Conditions	PCV Assay (%) ± RSD *		LYS Assay (%) ± RSD *	
	0 Month	6 Months	0 Month	6 Months
5 ± 2 °C	100.24 ± 0.55	98.60 ± 0.39	99.76 ± 0.37	97.91 ± 1.01
25 ± 2 °C	100.24 ± 0.55	98.53 ± 0.50	99.76 ± 0.37	98.69 ± 0.94
40 ± 2 °C	100.24 ± 0.55	97.84 ± 1.08	99.76 ± 0.37	97.72 ± 1.21

* RSD, relative standard deviation (n = 3).

## Supplementary Materials

The following are available online at https://www.mdpi.com/1996-1944/12/19/3154/s1, Figure S1: DSC curves of EDTA, PROPYL, METHYL, BHT, and PARAFF, Figure S2: DSC curves of LAURYL, POLAWAX, CET ALC, PROP GLY, and LIQ PET.

## References

[1] Hodge R.A.V. (1993). Famciclovir and Penciclovir. The Mode of Action of Famciclovir Including Its Conversion to Penciclovir. Antivir. Chem. Chemother..

[2] Boyd M.R., Safrin S., Kern E.R. (1993). Penciclovir: A review of its spectrum of activity, selectivity, and cross-resistance pattern. Antivir. Chem. Chemother..

[3] Mondal D. (2016). Penciclovir. Ref. Modul. Biomed. Sci..

[4] Abdel-Haq N., Chearskul P., Al-Tatari H., Asmar B. (2006). New antiviral agents. Indian J. Pediatr..

[5] Raborn G.W., Martel A.Y., Lassonde M., Lewis M.A.O., Boon R., Spruance S.L. (2002). Effective treatment of herpes simplex labialis with penciclovir cream: Combined results of two trials. J. Am. Dent. Assoc..

[6] Boyd M.R., Bacon T.H., Sutton D., Cole M. (1987). Antiherpesvirus activity of 9-(4-hydroxy-3-hydroxymethylbut-1-yl)guanine (BRL 39123) in cell culture. Antimicrob. Agents Chemother..

[7] Abdelhameed A.S., Bakheit A.H., Almutairi F.M., AlRabiah H., Kadi A.A. (2017). Biophysical and in silico studies of the interaction between the anti-viral agents acyclovir and penciclovir, and human serum albumin. Molecules.

[8] Kim D.-K., Lee N., Ryu S.H., Kim Y.-W., Kim J.S., Chang K., Im G.-J., Choi W.-S., Cho Y.-B., Kim K.H. (1999). Synthesis and evaluation of 2-Amino-9-(3-hydroxymethyl-4- alkoxycarbonyloxybut-1-yl)purines as potential prodrugs of penciclovir. Bioorg. Med. Chem..

[9] Zhu W., Yu A., Wang W., Dong R., Wu J., Zhai G. (2008). Formulation design of microemulsion for dermal delivery of penciclovir. Int. J. Pharm..

[10] Poole C.L., James S.H. (2018). Antiviral therapies for herpesviruses: Current agents and new directions. Clin. Ther..

[11] Brazil ANVISA—Lista de Medicamentos de Referência. http://portal.anvisa.gov.br/registros-e-autorizacoes/medicamentos/produtos/medicamentos-de-referencia/lista.

[12] Crimi S., Fiorillo L., Bianchi A., D’Amico C., Amoroso G., Gorassini F., Mastroieni R., Marino S., Scoglio C., Catalano F. (2019). Herpes Virus, Oral Clinical Signs and QoL: Systematic Review of Recent Data. Viruses.

[13] Tao M., Zhu M., Wu C., He Z. (2015). Degradation kinetic study of lysine in lysine hydrochloride solutions for injection by determining its main degradation product. Asian J. Pharm. Sci..

[14] Griffith R.S., Norins A.L., Kagan C. (1978). A multicentered study of lysine therapy in herpes simplex infection. Dermatologica.

[15] Gaby A.R. (2006). Natural remedies for herpes simplex. Altern. Med. Rev..

[16] Moreton C. Functionality and Performance of Excipients in a Quality-by-Design World. http://www.finnbrit.com/SubPages/Background/PDFFiles/QbDAPR_ExcipientSupplement2010.pdf.

[17] Zarmpi P., Flanagan T., Meehan E., Mann J., Fotaki N. (2017). Biopharmaceutical aspects and implications of excipient variability in drug product performance. Eur. J. Pharm. Biopharm..

[18] DiFeo T.J. (2003). Drug product development: A technical review of chemistry, manufacturing, and controls information for the support of pharmaceutical compound licensing activities. Drug Dev. Ind. Pharm..

[19] Mohamed A.I., Abd-Motagaly A.M.E., Ahmed O.A.A., Amin S., Ali A.I.M. (2017). Investigation of drug-polymer compatibility using chemometric-assisted UV-spectrophotometry. Pharmaceutics.

[20] Ford J.L., Timmins P. (1989). Pharmaceutical Thermal Analysis.

[21] Cunha-Filho M.S.S., Martínez-Pacheco R., Landín M. (2007). Compatibility of the antitumoral β-lapachone with different solid dosage forms excipients. J. Pharm. Biomed. Anal..

[22] Matos A.P.S., Costa J.S., Boniatti J., Seiceira R.C., Pitaluga Jr A., Oliveira D.L., Viçosa A.L., Holandino C. (2016). Compatibility study between diazepam and tablet excipients. J. Therm. Anal. Calorim..

[23] Sun B.-W., Fu Y., Chen L., Ding T., Zhai L.-H. (2017). Compatibility study of rivaroxaban and its pharmaceutical excipients. J. Anal. Calorim..

[24] Veiga A., Oliveira P.R., Bernardi L.S., Mendes C., Silva M.A.S., Sangoi M.S., Janissek P.R., Murakami F.S. (2018). Solid-state compatibility studies of a drug without melting point: The case of omeprazole sodium. J. Therm. Anal. Calorim..

[25] Sultana S., Mohammed S. (2017). A review on stability studies of pharmaceutical products. Int. J. Pharm. Biol. Res..

[26] Rowe R.C., Sheskey P.J., Quinn M.E. (2009). Handbook of Pharmaceutical Excipients.

[27] Gomes EC de L., Ercole de Carvalho I., Fialho S.L., Barbosa J., Yoshida M.I., da Silva Cunha Júnior A. (2018). Mixing method influence on compatibility and polymorphism studies by DSC and statistical analysis. J. Anal. Calorim..

[28] Ferreira A.O., Brandão M.F., Silva M.A.D.C.G. (2002). Guia Prático para Farmácia de Manipulação.

[29] Ribeiro C. (2010). Cosmetologia Aplicada a Dermoestética.

[30] (2003). International Conference on Harmonization, ICH Harmonised Tripartite Guideline: Stability Testing of New Drug Substances and Products Q1A (R2).

[31] Ahmed A., Barry B.W., Williams A.C., Davis A.F. (2004). Penciclovir solubility in Eudragit films: A comparison of X-ray, thermal, microscopic and release rate techniques. J. Pharm. Biomed. Anal..

[32] Aydin M., Kartal Z., Osmanoǧlu Ş., Halim Başkan M., Topkaya R. (2011). EPR and FT-IR Spectroscopic Studies of L-Lysine Monohydrochloride and L-Glutamic Acid Hydrochloride Powders. J. Mol. Struct..

[33] Kasten G., Nouri K., Grohganz H., Rades T., Löbmann K. (2017). Performance comparison between crystalline and co-amorphous salts of indomethacin-lysine. Int. J. Pharm..

[34] Harnden M.R., Jarvest R.L., Slawin A.M.Z., Williams D.J. (1990). Crystal and molecular structures of the antiviral acyclonucleoside 9-[4-hydroxy-3-(hydroxymethyl)butyl]guanine (brl 39123, Penciclovir) and its Prodrug 9-[4-acetoxy-3-(acetoxymethyl)butyl]-2-aminopurine (brl 42810, Famciclovir). J. Nucleos. Nucleot..

[35] Batista J.C. (October 2016). Study of the Vibrational and Structural Properties of Crystals L-Lysine Dihydrate Monohydrochloride and DL-Lysine Monohydrochloride. Ph.D. Thesis.

[36] Williams P.A., Hughes C.E., Harris K.D.M. (2015). L-Lysine: Exploiting Powder X-ray Diffraction to Complete the Set of Crystal Structures of the 20 Directly Encoded Proteinogenic Amino Acids. Angew. Chem.-Int. Ed..

[37] Phadnis N.V., Cavatur R.K., Suryanarayanan R. (1997). Identification of drugs in pharmaceutical dosage forms by X-ray powder diffractometry. J. Pharm. Biomed. Anal..

[38] Barbosa LC de A. (2007). Infrared Spectroscopy in the Characterization of Organic Compounds.

[39] Garoufis A., Karidi K., Hadjiliadis N., Kasselouri S., Kobe J., Balzarini J., De Clercq E. (2001). Synthesis, Characterization and Antiviral Properties of Pd(II) Complexes with Penciclovir. Met. Based Drugs.

[40] Akimsheva E.Y., Dolinina E.S., Parfenyuk E.V. (2019). Interactions of Sol-Gel Encapsulated Acyclovir with Silica Matrix. Colloid. Surf. B.

[41] Petrosyan A.M., Ghazaryan V.V. (2009). Vibrational spectra of L-Lysine monohydrochloride dihydrate and its two anhydrous forms. J. Mol. Struct..

[42] Bruni G., Berbenni V., Milanese C., Girella A., Marini A. (2010). Drug-excipient compatibility studies in binary and ternary mixtures by physico-chemical techniques. J. Anal. Calorim..

[43] Neto H.S., Novák C., Matos J.R. (2009). Thermal analysis and compatibility studies of prednicarbate with excipients used in semi solid pharmaceutical form. J. Anal. Calorim..

[44] Gelbrich T., Braun D.E., Ellern A., Griesser U.J. (2013). Four polymorphs of methylparaben: Structural relationships and relative energy differences. Cryst. Growth Des..

[45] Moyano M.A., Broussalis A.M., Segall A.I. (2010). Thermal analysis of lipoic acid and evaluation of the compatibility with excipientes. J. Anal. Calorim..

[46] de Oliveira M.A., Yoshida M.I., Silva D.C. (2014). Quality evaluation of pharmaceutical formulations containing hydrochlorothiazide. Molecules.

[47] Reddy S.T., Sivaramakrishna D., Swamy M.J. (2017). Physicochemical characterization of Lauryl Glycinate-Dodecyl sulfate equimolar complex: A base-triggerable catanionic liposomal system. Colloid. Surf. A.

[48] Lira A.M., Araújo A.A.S., Basílio I.D.J., Santos B.L.L., Santana D.P., Macedo R.O. (2007). Compatibility studies of lapachol with pharmaceutical excipients for the development of topical formulations. Thermochim. Acta.

[49] Wong L.P., Gilligan C.A., Po A.L.W. (1992). Preparation and Characterisation of Sustained-Release Ibuprofen-Cetostearyl Alcohol Spheres. Int. J. Pharm..

[50] Batista RS de A. (March 2015). Development of Analytical Methodology for Analysis of Thermal Stability of Formulation Retinoic Acid Cream. Master’s Thesis.

[51] de Cássia da Silva R., Tadeu Gomes Cavalheiro É. (2015). Synthesis, characterization, and thermal analysis of alginate and monoethanolamine product. J. Therm. Anal. Calorim..

[52] Narang A.S., Yamniuk A., Zhang L., Comezoglu S.N., Bindra D.S., Varia S.A., Doyle M., Badawy S., Narang A.S., Boddu S.H. (2015). Excipient Applications in Formulation Design and Drug Delivery.

