# Supplementary material for: Solid-State Characterization and Compatibility Studies of Penciclovir, Lysine Hydrochloride, and Pharmaceutical Excipients
Source: Materials (Basel). 2019 Sep 27;12(19):3154. doi: 10.3390/ma12193154 (PMC6803830; doi:10.3390/ma12193154)
Supplement: Supplementary file 1 [file materials-12-03154-s001.pdf]

materials

Article

Solid-State Characterization and Compatibility Studies of Penciclovir, Lysine Hydrochloride, and Pharmaceutical Excipients

Rafaela Z. C. Meira [1], Isabela F. B. Biscaia [1], Camila Nogueira [1], Fabio S. Murakami [2], Larissa S. Bernardi [1] and Paulo R. Oliveira [1,*]

Supplementary Materials

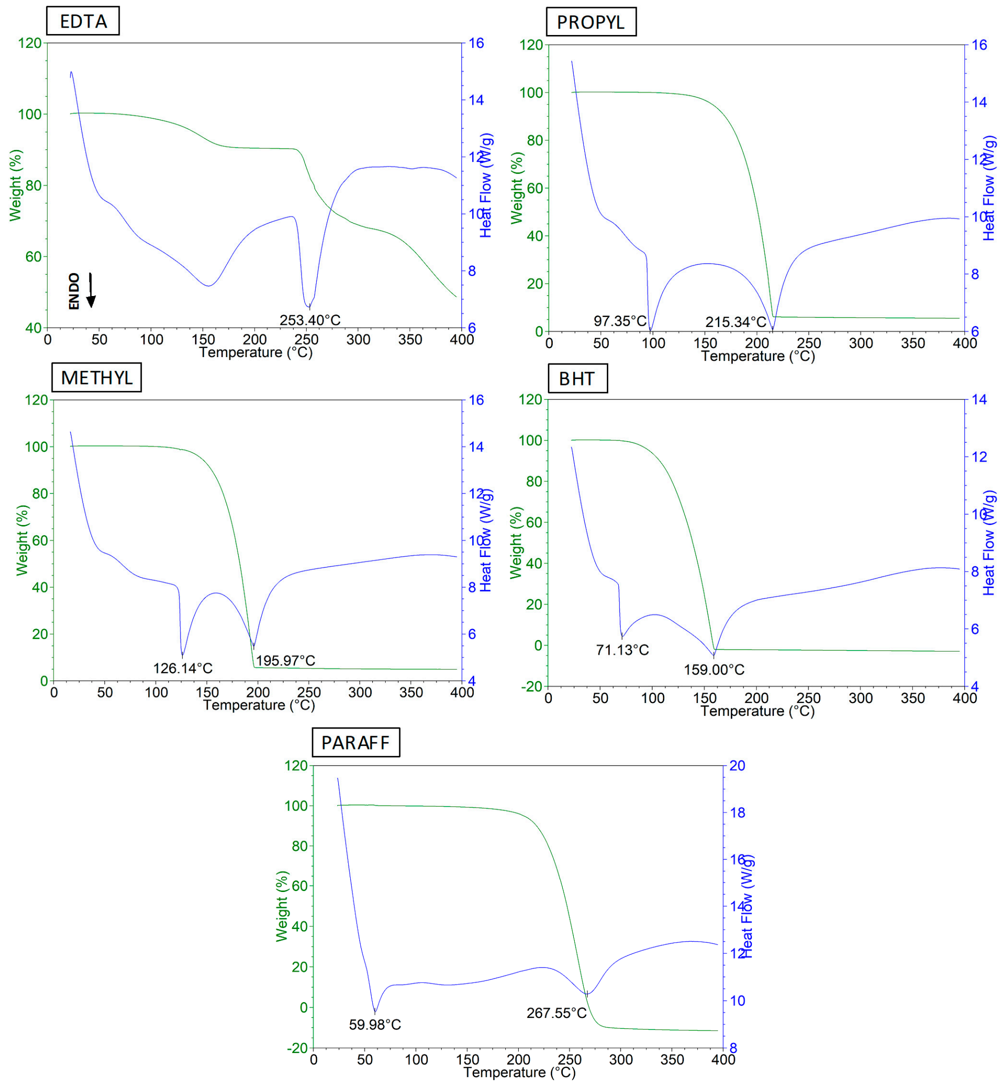

Figure S1. DSC curves of EDTA, PROPYL, METHYL, BHT, and PARAFF.

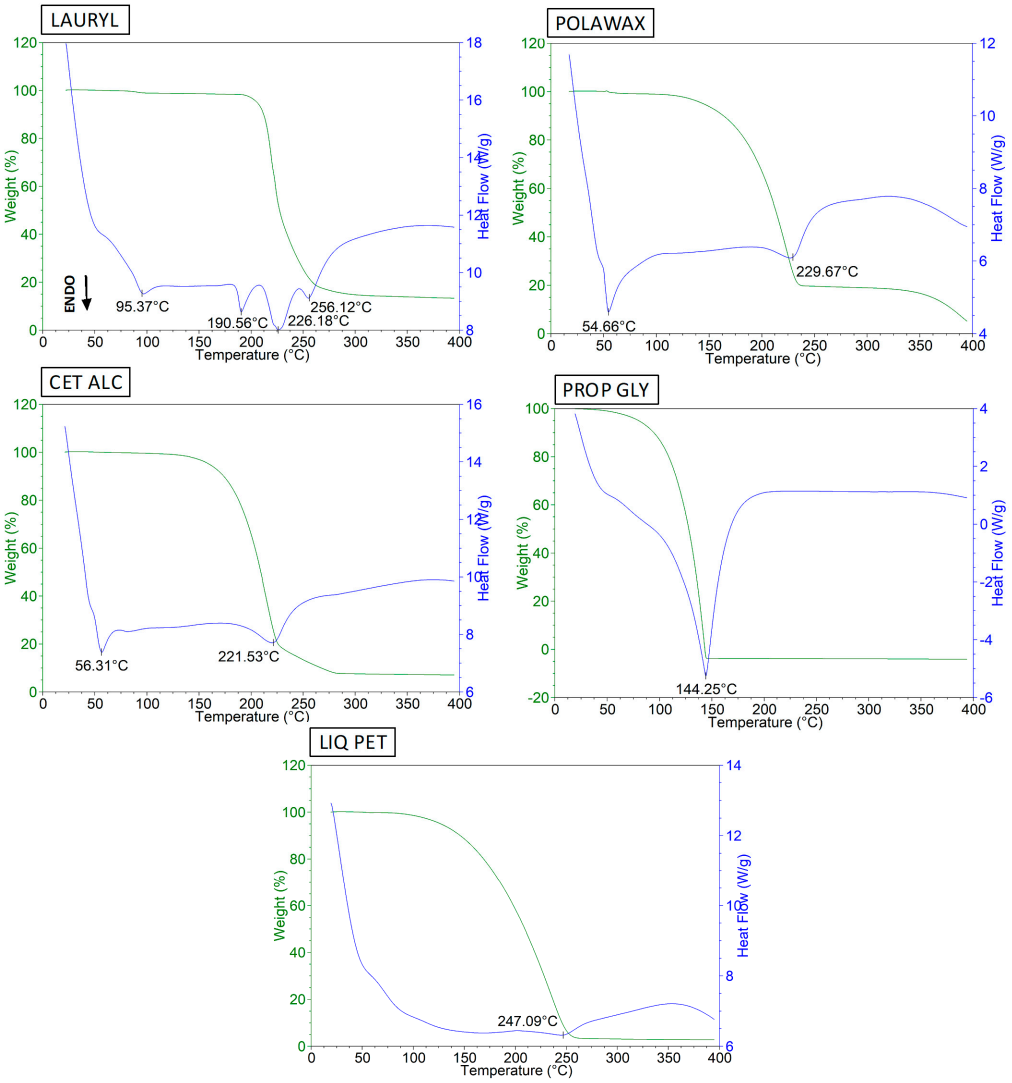

Figure S2. DSC curves of LAURYL, POLAWAX, CET ALC, PROP GLY, and LIQ PET.
